# Anti-Inflammatory Activity of Kurarinone Involves Induction of HO-1 via the KEAP1/Nrf2 Pathway

**DOI:** 10.3390/antiox9090842

**Published:** 2020-09-09

**Authors:** Sakiko Nishikawa, Yasumichi Inoue, Yuka Hori, Chiharu Miyajima, Daisuke Morishita, Nobumichi Ohoka, Shigeaki Hida, Toshiaki Makino, Hidetoshi Hayashi

**Affiliations:** 1 Department of Cell Signaling, Graduate School of Pharmaceutical Sciences, Nagoya City University, Nagoya 467-8603, Japan; sakikow5@gmail.com (S.N.); yukahori8123@gmail.com (Y.H.); miyajima@phar.nagoya-cu.ac.jp (C.M.); daisuke.b.m.1215@gmail.com (D.M.); 2 Department of Innovative Therapeutic Sciences, Cooperative Major in Nanopharmaceutical Sciences, Graduate School of Pharmaceutical Sciences, Nagoya City University, Nagoya 467-8603, Japan; 3 Division of Molecular Target and Gene Therapy Products, National Institute of Health Sciences, Kanagawa 210-9501, Japan; n-ohoka@nihs.go.jp; 4 Department of Molecular and Cellular Health Sciences, Graduate School of Pharmaceutical Sciences, Nagoya City University, Nagoya 467-8603, Japan; hida@phar.nagoya-cu.ac.jp; 5 Department of Pharmacognosy, Graduate School of Pharmaceutical Sciences, Nagoya City University, Nagoya 467-8603, Japan; makino@phar.nagoya-cu.ac.jp

**Keywords:** anti-inflammatory, antioxidant, HO-1, KEAP1, kurarinone, Nrf2, *Sophora flavescens*

## Abstract

Kurarinone, a flavonoid isolated from the roots of *Sophora flavescens*, was suggested to exert potent antioxidant and immunosuppressive effects. However, the underlying mechanisms remain unclear. Nuclear factor erythroid 2-related factor 2 (Nrf2) is a key transcription factor that regulates the antioxidant defense system with anti-inflammatory activity. In the present study, we demonstrated that kurarinone activated Nrf2 and increased the expression of antioxidant enzymes, including heme oxygenase-1 (HO-1). Mechanistically, kurarinone downregulated the expression of kelch-like ECH-associated protein 1 (KEAP1), subsequently leading to the activation of Nrf2. Kurarinone also inhibited the expression of the inflammatory cytokine, interleukin (IL)-1β, and inducible nitric oxide synthase (iNos) in lipopolysaccharide (LPS)-stimulated RAW264.7 macrophages. The overexpression of HO-1 suppressed the LPS-induced production of inflammatory mediators in RAW264.7 cells, and the immunosuppressive effects of kurarinone were partially inhibited by a treatment with Tin Protomorphyrin IX (TinPPIX), an inhibitor of HO-1. These results indicate that kurarinone activates the KEAP1/Nrf2 pathway to induce HO-1 expression, thereby exerting immunosuppressive effects.

## 1. Introduction

**Figure 1:**
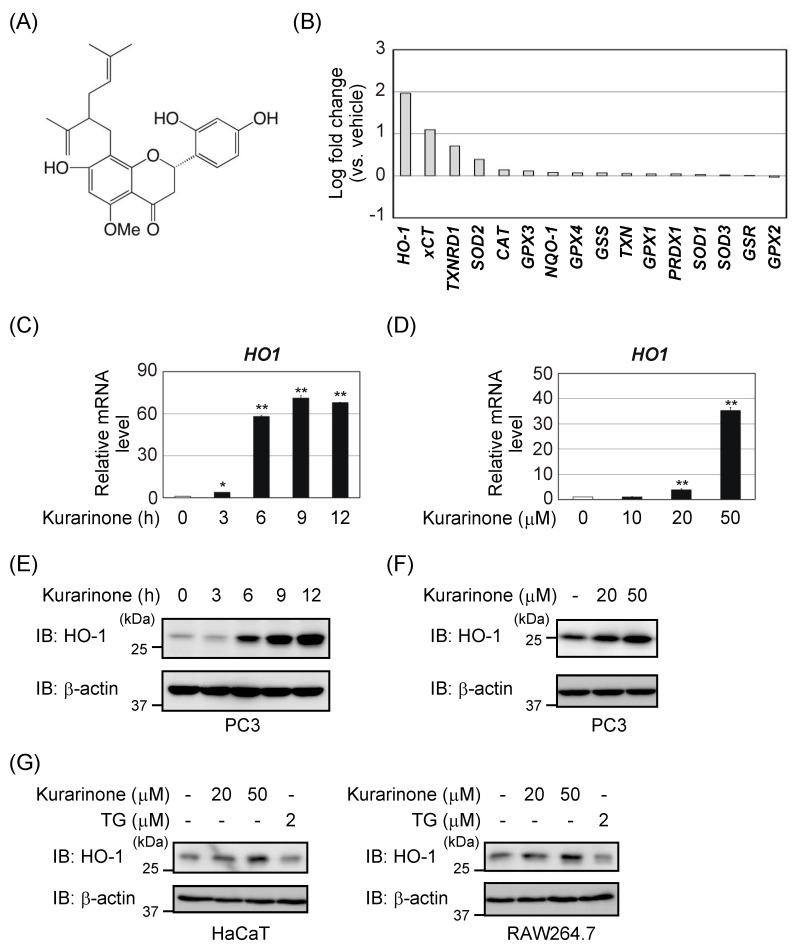
Kurarinone induces several antioxidant enzymes, including HO-1. (**A**) Structure of kurarinone. (**B**) PC3 cells were treated with 50 μM kurarinone for 6 h. The expression of each gene was assessed by quantitative PCR (qPCR). The experiment was performed in duplicates. Results show fold changes from the DMSO-treated control group. (**C**,**E**) PC3 cells were treated with 50 μM kurarinone for the indicated periods. (**C**) *HO-1* mRNA expression levels were assessed by qPCR. Results were shown as the mean ± S.D. (*n* = 3). (**E**) Cell lysates were immunoblotted with the indicated antibodies. (**D**,**F**) PC3 cells were treated with the indicated doses of kurarinone for 6 h. (**D**) *HO-1* mRNA expression levels were assessed by qPCR, as in (**C**). (**F**) Cell lysates were immunoblotted with the indicated antibodies, as in (**E**). (**G**) RAW264.7 and HaCaT cells were treated with the indicated doses of kurarinone or 2 μM thapsigargin (TG) for 6 h. Cell lysates were immunoblotted with the indicated antibodies, as in (**E**). Significant differences were indicated as ** *p* < 0.01, * *p* < 0.05 vs. the group without kurarinone-treatment.

The roots of *Sophora flavescens* (Fabaceae), also known as “Kushen” in Chinese and “Kujin” in Japanese, were traditionally used in combination with other herbal medicines to treat asthma, bronchitis, bacterial and fungal infections, skin disorders, and inflammation [1]. Many prenylated flavonoids with a number of biological activities were isolated from *S. flavescens* [2]. Kurarinone, a lavandulyl flavanone, is abundant in the roots of *S. flavescens* (Figure 1A) [3,4]. It has several beneficial biological properties, including antioxidant [2] and anti-inflammatory activities [5]. Although kurarinone was shown to inhibit the activity of nuclear factor kappa B (NF-κB) [6], but its mechanisms of action remain unclear.

The transcription factor NF-E2-related factor (Nrf2) plays a central role in the oxidative stress response pathway [7]. Under unstressed conditions, kelch-like ECH-associated protein 1 (KEAP1), which forms part of the E3 ligase, binds to cytoplasmic Nrf2 and promotes the ubiquitination and proteasome-dependent degradation of Nrf2. In response to stress, Nrf2 dissociates from KEAP1 and then stabilizes and accumulates in the nucleus. Nrf2 induces the expression of a series of antioxidant and detoxification enzyme genes (e.g., heme-oxygenase 1 (HO-1), system xc^−^ (xCT), and NAD(P)H:quinone oxidoreductase 1 (NQO1)), and activates a wide range of cellular defense mechanisms, thereby enhancing the overall capacity of the cells to detoxify and eliminate toxic substances [8].

HO-1 is an important antioxidant protein that is regulated by Nrf2. It is an inducible enzyme that decomposes oxidative and harmful free heme into carbon monoxide (CO), free iron, and biliverdin, which are then converted into bilirubin [9]. CO exerts anti-apoptotic and anti-inflammatory effects [10], and bilirubin exhibits strong antioxidant activity [11]. These protective effects suggest that HO-1 plays important roles in reducing inflammation [12]. Therefore, pharmacologically increasing the expression of HO-1 is currently considered to be a novel targeted therapy [13]. In addition, Nrf2 directly suppresses inflammatory responses. Nrf2 specifically blocks the pro-inflammatory transcriptional program mediated by NF-κB [14]. Therefore, the pharmacological activation of Nrf2 is a promising therapeutic approach for several chronic diseases, including multiple sclerosis [15]. The utility of Nrf2 activators in the treatment of diabetic nephropathy and pulmonary hypertension was also suggested, and they are currently undergoing clinical trials [16].

In the present study, we demonstrated that kurarinone downregulated the expression of KEAP1, leading to the activation of Nrf2. We also showed that the anti-inflammatory effects of kurarinone were due to the activation of the Nrf2/HO-1 axis. Therefore, kurarinone might be used for clinical applications and offers new therapeutic options for chronic inflammatory diseases.

## 2. Materials and Methods

### 2.1. Cell Lines, Plasmids, and RNA Interference

RAW264.7 and HaCaT cells were maintained in Dulbecco’s modified Eagle’s medium (Sigma, St. Louis, MO, USA), supplemented with 4.5 g/L glucose, 10% fetal bovine serum (FBS) (Sigma), 100 U/mL of penicillin G, and 100 μg/mL of streptomycin, as previously described [17]. PC3 cells were cultured in the Roswell Park Memorial Institute 1640 medium (Sigma) containing 10% FBS and penicillin/streptomycin [18]. Cells were grown in a 5% CO_2_ atmosphere at 37 °C.

cDNA encoding human HO-1 was amplified by PCR and cloned into the lentivirus vector CSIIEF/FLAG [19], which has an elongation factor gene promoter for protein expression in mammalian cells. The correct sequence of the cloned expression vector was confirmed by DNA sequencing. RAW264.7 cells were infected with viral particles, according to standard protocols [20].

In short interfering RNA (siRNA) transfection, siRNAs were transfected using Lipofectamine RNAiMAX reagent (Invitrogen, Carlsbad, CA, USA), according to the manufacturer’s protocol. RAW264.7 cells were transfected with siRNAs through nucleofection. Two million RAW264.7 cells were suspended in buffer provided in Nucleofection Kit V (Lonza, Basel, Switzerland), mixed with siRNA (30 pmol), and electroporated with the D-032 program of Nucleofector IIS (Lonza), as suggested by the manufacturer. Human *Nrf2* siRNA #1 (NM_006164.4; sense: 5′-CGUUUGUAGAUGACAAUGA-3′) and human *Nrf2* siRNA #2 (NM_006164.4; sense: 5′-GAAUGGUCCUAAAACACCA-3′) were purchased from Ambion (Austin, TX, USA). Mouse *Nrf2* siRNA #1 (NM_010902.4; sense: 5′-UGUUUGACUUUAGUCAGCGACAGAA-3′), mouse *Nrf2* siRNA #2 (NM_010902.4; sense: 5′-GCAUGUUACGUGAUGAGGAUGGAAA-3′), and Stealth RNAi™ siRNA Negative Control Med GC Duplex were also obtained from Invitrogen.

### 2.2. RNA Extraction, Reverse Transcription (RT), and Quantitative PCR (qPCR)

Total RNA extraction was performed, as reported previously [21]. First-strand cDNA was synthesized with the PrimeScript first-strand cDNA Synthesis Kit (TaKaRa Bio Inc., Shiga, Japan), as described elsewhere [22]. qPCR was performed according to a previously described method [22]. The specificities of the detected signals were confirmed by a dissociation curve, which consisted of a single peak. Values were normalized by *β-actin*. The primer sequences are listed in Table 1.

### 2.3. Immunochemical Methods and Antibodies

Immunoblotting was performed as previously described [23]. Cytoplasmic and nuclear extracts were prepared using NE-PER Nuclear and Cytoplasmic Extraction Reagents (Thermo Fisher Scientific, Waltham, MA, USA). The following commercially available antibodies were used—anti-HO-1 (10701-1-AP; ProteinTech, Chicago, IL, USA), anti-Nrf2 (#12721; Cell Signaling Technology, Beverly, MA, USA), anti-KEAP1 (#4617; Cell Signaling Technology), anti-FLAG (M2; Sigma), anti-β-actin (AC-15; Sigma), anti-histone H4 (MABI0400; MBL, Nagoya, Japan), anti-p62 (PM045; MBL), and anti-Hsp90 (Clone 68/Hsp90; BD Biosciences, Franklin Lakes, NJ, USA).

### 2.4. Chromatin Immunoprecipitation (ChIP) Assay

Cells were crosslinked with 1% formaldehyde and then lysed in SDS lysis buffer (50 mM Tris-HCl, pH 8.0, 1% SDS, 10 mM EDTA, and protease inhibitors). The ChIP procedure was performed as previously described [24]. Primer sequences are listed in Table 2.

### 2.5. Cell Viability Assay

Cell viability was assessed using WST-8, according to the manufacturer’s instructions (Dojindo, Kumamoto, Japan). Cells were seeded at a concentration of 5 × 10^3^ cells per well on a 96-well plate. After 24 h, the cells were treated with kurarinone for 24 h. The WST-8 reagent was added and the cells were incubated at 37 °C for 3 h, in a humidified atmosphere of 5% CO_2_. The absorbance at 450 nm of the medium was measured [18].

### 2.6. Chemicals

Kurarinone was prepared as previously reported [18]. The identity and purity of kurarinone was confirmed by LC-MS and NMR analyses [18]. Thapsigargin (TG) was purchased from Fujifilm-Wako (Osaka, Japan). Tin Protomorphyrin IX (TinPPIX) and ML385 were purchased from Cayman Chemical (Ann Arbor, MI, USA). Lipopolysaccharide (LPS) and other chemicals were purchased from Sigma.

### 2.7. Statistical Tests

The significance of differences between the two groups was evaluated using the two-tailed Student’s *t*-test. In multi-group analyses, significance was assessed using a one-way ANOVA with the post hoc Tukey-Kramer HSD test.

## 3. Results

### 3.1. Kurarinone Induces Several Antioxidant Enzymes, Including HO-1

We examined the effects of kurarinone on the mRNA expression levels of antioxidant enzymes, including superoxide dismutase (SOD), HO-1, glutathione peroxidase (GPX), and catalase (CAT), in human prostate cancer PC3 cells. As shown in Figure 1B, the treatment of PC3 cells with kurarinone increased the mRNA expression levels of several antioxidant enzymes. It significantly increased *HO-1* mRNA expression levels in PC3 cells. Kurarinone dose- and time-dependently increased HO-1 expression levels in PC3 cells (Figure 1C–F). The induction of HO-1 expression was also observed in HaCaT cells (human keratinocytes) and RAW264.7 cells (mouse macrophages) (Figure 1G).

### 3.2. Kurarinone Induces HO-1 Expression in an Nrf2-Dependent Manner

**Figure 2:**
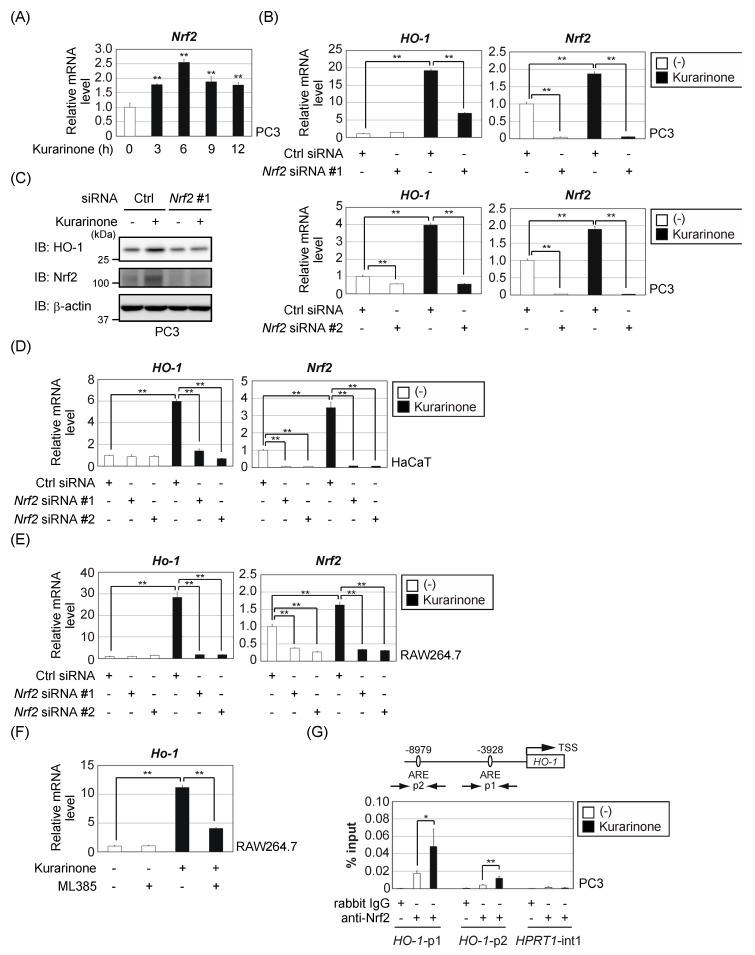
Kurarinone induces HO-1 expression in an Nrf2-dependent manner. (**A**) PC3 cells were treated with 50 μM kurarinone for the indicated periods. The expression of *Nrf2* mRNA was assessed by qPCR. Results were shown as the mean ± S.D. (*n* = 3). Significant differences are indicated as ** *p* < 0.01 vs. the group without kurarinone-treatment. (**B**,**C**) PC3 cells were transiently transfected with the indicated siRNAs. After 48 h, the cells were treated with 50 μM kurarinone for 6 h. (**B**) The expression level of each gene was assessed by qPCR. Results were shown as the mean ± S.D. (*n* = 3). Significant differences are indicated as ** *p* < 0.01. (**C**) Cell lysates were immunoblotted with the indicated antibodies. Ctrl, control. (**D**) HaCaT cells were transiently transfected with the indicated siRNAs. After 48 h, the cells were treated with 50 μM kurarinone for 6 h. The expression level of each gene was assessed by qPCR, as in (**B**). (**E**) RAW264.7 cells were electroporated with the indicated siRNAs. After 48 h, the cells were treated with 50 μM kurarinone for 12 h. The expression level of each gene was assessed by qPCR, as in (**B**). (**F**) RAW264.7 cells were pretreated with 10 μM ML385 for 1 h and then treated with 50 μM kurarinone for 12 h. The expression level of each gene was assessed by qPCR, as in (**B**). (**G**) PC3 cells were treated with 50 μM kurarinone for 6 h. Cell lysates were subjected to a chromatin immunoprecipitation (ChIP) analysis with the indicated antibodies. Extracted DNA fragments were analyzed by qPCR. Results were shown as the mean ± S.D. (*n* = 3). Significant differences are indicated as ** *p* < 0.01, * *p* < 0.05.

HO-1 is a representative Nrf2 target gene product [7,8]. We investigated the involvement of Nrf2 in the induction of HO-1 by kurarinone. The treatment of PC3 cells with kurarinone, significantly increased the *Nrf2* mRNA levels (Figure 2A). To establish whether Nrf2 is required for the induction of HO-1 by kurarinone, PC3 cells were treated with siRNA to deplete Nrf2. As shown in Figure 2B,C, the knockdown of Nrf2, reduced the induction of HO-1 expression by kurarinone. Depletion of Nrf2 also suppressed kurarinone-induced *HO-1* mRNA expression in HaCaT cells (Figure 2D) and RAW264.7 cells (Figure 2E). Moreover, kurarinone-induced *Ho-1* mRNA expression was attenuated by ML385 (Figure 2F), an Nrf2 inhibitor that binds to Nrf2 and inhibits its target gene expression [25]. These results indicate that Nrf2 is responsible for the upregulated expression of HO-1 by kurarinone.

Nrf2 regulates gene expression by binding to the antioxidant response elements (ARE) on the promoters of its target genes [7,8]. In the human *HO-1* gene locus, Nrf2 interacts with two sites containing multiple ARE motifs—a more proximal site located at −3928 bp, upstream of the transcription start site (p1) and a more distal site located at −8979 bp upstream (p2) (Figure 2G) [26]. ChIP analyses demonstrated that the kurarinone treatment induced Nrf2 binding to both of these sites.

### 3.3. Kurarinone-Induced KEAP1 Downregulation Contributes to the Activation of Nrf2

**Figure 3:**
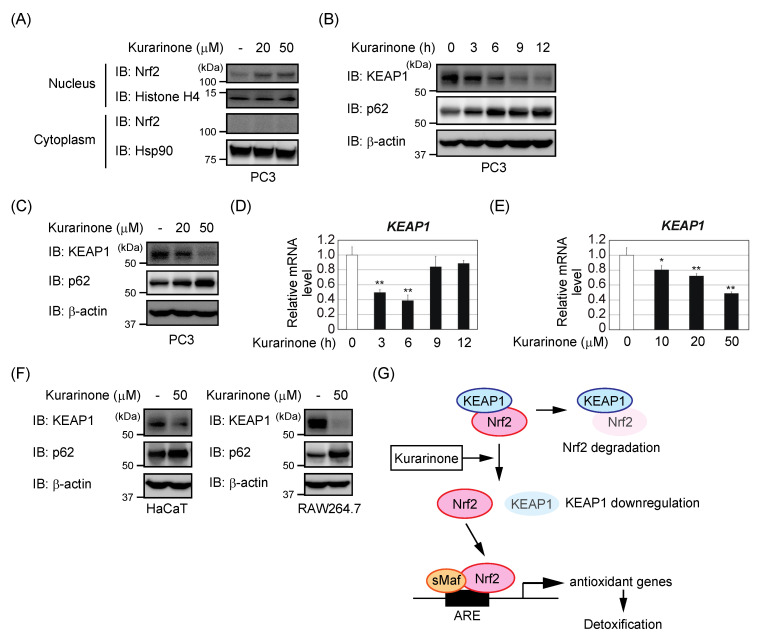
Kurarinone-induced KEAP1 downregulation contributes to the activation of Nrf2. (**A**) After treating the PC3 cells with the indicated doses of kurarinone for 6 h, nuclear and cytoplasmic extracts were subjected to immunoblotting with the indicated antibodies. (**B**,**D**) PC3 cells were treated with 50 μM kurarinone for the indicated periods. (**B**) Cell lysates were immunoblotted with the indicated antibodies. (**D**) The expression of *KEAP1* mRNA was assessed by qPCR. The results were shown as the mean ± S.D. (*n* = 3). (**C**,**E**) PC3 cells were treated with the indicated doses of kurarinone for 6 h. (**C**) Cell lysates were immunoblotted with the indicated antibodies, as in (**B**). (**E**) The expression of *KEAP1* mRNA was assessed by qPCR, as in (**D**). Significant differences are indicated as ** *p* < 0.01, * *p* < 0.05 vs. the group without kurarinone-treatment. (**F**) HaCaT and RAW264.7 cells were treated with the indicated doses of kurarinone for 6 h. Cell lysates were immunoblotted with the indicated antibodies, as in (**B**). (**G**) Kurarinone downregulates KEAP1 expression, which might lead to the activation of Nrf2. In the nucleus, Nrf2 binds to small musculoaponeurotic fibrosarcoma (sMaf) as a partner, to activate the specific antioxidant responsive elements (ARE) present in gene promoters. It then elicits the transcription of several antioxidant genes, including heme-oxygenase 1 (HO-1), which induce detoxification.

We examined Nrf2 protein levels in cytoplasmic and nuclear extracts of PC3 cells, treated with different concentrations of kurarinone for 6 h. As shown in Figure 3A, the accumulation of Nrf2 in the nucleus was observed in PC3 cells treated with kurarinone. On the other hand, the Nrf2 protein was not detected in the cytoplasmic extracts, in the presence or absence of kurarinone.

Since KEAP1 suppresses the accumulation of Nrf2 in the nucleus, we investigated whether the kurarinone treatment affected the expression of the KEAP1 protein. Kurarinone markedly reduced KEAP1 protein levels in PC3 cells in time- and dose-dependent manners (Figure 3B,C). The qRT-PCR analysis revealed that *KEAP1* mRNA levels were reduced by the kurarinone treatment, reaching approximately 50% after 6 h, and then recovered (Figure 3D). As shown in Figure 3E, the treatment with kurarinone for 6 h resulted in a dose-dependent decrease in the expression of *KEAP1* mRNA in PC3 cells. Moreover, the decreased expression of KEAP1 was also observed in the HaCaT cells and RAW264.7 cells (Figure 3F). These results suggest that kurarinone downregulates the KEAP1 expression, leading to the activation of Nrf2 (Figure 3G).

The treatment with kurarinone resulted in a transient decrease in the *KEAP1* mRNA levels, but a persistent decrease in the KEAP1 protein levels (Figure 3B,D). Therefore, the mechanism underlying the decrease in KEAP1 protein expression by kurarinone at a later stage, did not appear to involve a decrease in *KEAP1* mRNA levels. p62 is a target gene of Nrf2, and might contribute to the activation of Nrf2 by forming a complex with KEAP1 and competing with Nrf2 for binding with KEAP1 [27,28]. We confirmed the expression of the p62 protein after the kurarinone treatment in PC3 cells. As shown in Figure 3B,C, kurarinone dose- and time-dependently increased the p62 expression levels in PC3 cells. Therefore, the induction of p62 appeared to contribute to the kurarinone-induced decrease in KEAP1 expression. Further investigations are needed to elucidate this regulatory mechanism in more detail.

### 3.4. Kurarinone Suppressed the Expression of Il-1β and iNos in LPS-Stimulated RAW264.7 Macrophages by Upregulating the Expression of HO-1

**Figure 4:**
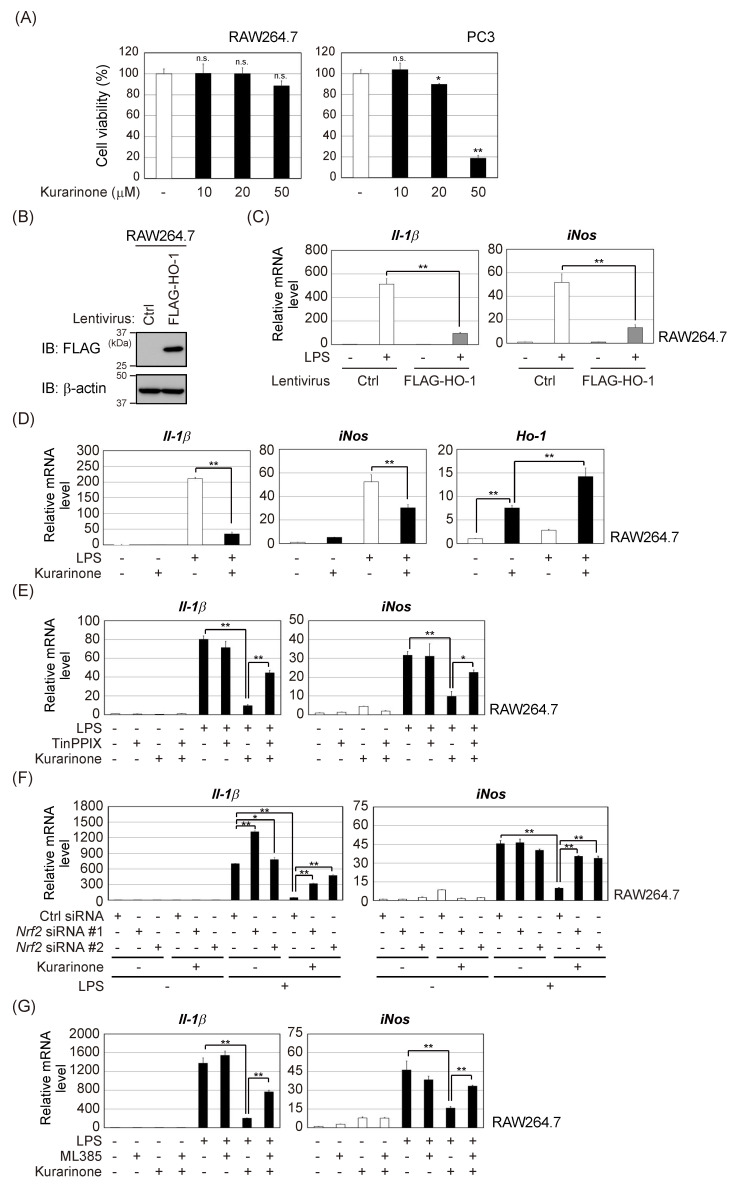
Kurarinone suppressed the expression of *Il-1β* and *iNos* mRNA in LPS-stimulated RAW264.7 macrophages by up-regulating HO-1 expression. (**A**) RAW264.7 and PC3 cells were exposed to the indicated doses of kurarinone for 24 h. Cell viability was measured using the WST-8 cell proliferation assay. Results were shown as the mean ± S.D. (*n* = 3). Significant differences are indicated as ** *p* < 0.01, * *p* < 0.05 vs. the group without kurarinone-treatment. n.s.: not significant. (**B**) RAW264.7 cells and RAW264.7/FLAG-HO-1 cells were subjected to immunoblotting with the indicated antibodies. Ctrl, control (**C**) RAW264.7 and RAW264.7/FLAG-HO-1 cells were treated with 100 ng/mL of LPS for 6 h. The expression level of each gene was assessed by qPCR. Results represent the mean ± S.D. (*n* = 3). (**D**) RAW264.7 cells were pretreated with 50 μM kurarinone for 6 h and then incubated with 100 ng/mL of LPS for 6 h. The expression level of each gene was assessed by qPCR. Results are shown as the mean ± S.D. (*n* = 3). (**E**) RAW264.7 cells were pretreated with 50 μM kurarinone and/or 10 μM TinPPIX for 6 h and then incubated with 100 ng/mL of LPS for 6 h. The expression level of each gene was assessed by qPCR, as in (**C**). (**F**) RAW264.7 cells were electroporated with the indicated siRNAs. After 48 h, cells were pretreated with 50 μM kurarinone for 6 h and then incubated with 100 ng/mL of LPS for 6 h. The expression level of each mRNA was assessed by qPCR, as in (**C**). (**G**) RAW264.7 cells were pretreated with 10 μM ML385 for 1 h, followed by 50 μM kurarinone treatment for 6 h. The cells were then incubated with 100 ng/mL of LPS for 6 h. The expression level of each mRNA was assessed by qPCR, as in (**C**). Significant differences are indicated as ** *p* < 0.01, * *p* < 0.05.

We investigated whether kurarinone-induced HO-1 expression was associated with the anti-inflammatory effects of kurarinone. We performed cell viability studies to assess the cytotoxic effects of kurarinone on RAW264.7 cells. As shown in Figure 4A, the cytotoxicity of kurarinone up to a concentration of 50 μM in RAW264.7 cells was negligible. On the other hand, the treatment with kurarinone resulted in decreased cell viability in PC3 cells, consistent with previous report [18]. Furthermore, we confirmed that exposure to LPS for 6 h significantly upregulated the mRNA expression of *interleukin (Il)-1β* and *inducible nitric oxide synthase (iNos)* in RAW264.7 cells. However, the overexpression of HO-1, suppressed these increases (Figure 4B,C). We also examined the effects of kurarinone on the induction of *Il-1β* and *iNos* mRNA expression in response to LPS. RAW264.7 cells were treated with kurarinone for 6 h and then with LPS for 6 h. We found that LPS-induced *Il-1β* and *iNos* mRNA expression was markedly suppressed by the kurarinone treatment (Figure 4D). HO-1 was previously shown to be induced by LPS via the activation of NF-κB [29], and its mRNA expression was further increased by a co-treatment with LPS and kurarinone.

**Figure 5:**
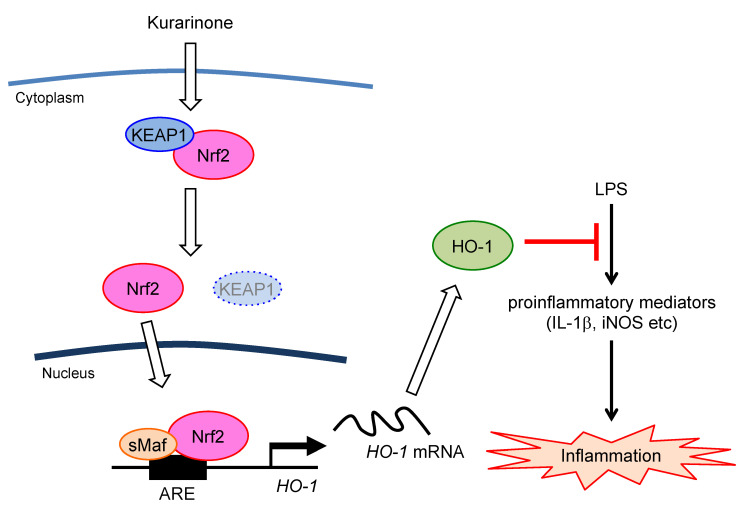
Schematic illustration showing the signal transduction pathway associated with the anti-inflammatory effects of kurarinone on LPS-stimulated RAW264.7 macrophage cells. Kurarinone induces the nuclear accumulation of Nrf2 and upregulates HO-1 protein expression, which attenuates the LPS-mediated production of proinflammatory mediators.

To clarify whether HO-1 is involved in the suppressive effects of kurarinone on LPS-induced proinflammatory responses, TinPPIX, a potent inhibitor of HO-1, was used. As shown in Figure 4E, the TinPPIX treatment markedly restored the kurarinone-mediated suppression of the LPS-induced upregulation of *Il-1β* and iNos mRNA. Furthermore, both Nrf2 knockdown or Nrf2 inhibitor ML385 also suppressed anti-inflammatory activities of kurarinone in RAW264.7 cells (Figure 4F,G). Collectively, the present results suggest that kurarinone suppresses the production of IL-1β and iNOS, by activating the Nrf2/HO-1 pathway and exerts anti-inflammatory effects (Figure 5).

## 4. Discussion

In the present study, we showed that kurarinone induced several antioxidant enzymes. We focused on HO-1, the expression of which was strongly induced, and confirmed that Nrf2 was involved in its induction. HO-1 exerts anti-inflammatory and antioxidant effects [9]. Kurarinone also exerts anti-inflammatory effects, and the underlying mechanism was suggested to involve the suppression of the activation of NF-κB [6]. We speculated that the induction of HO-1 by kurarinone might be involved in the anti-inflammatory effects of kurarinone. The pretreatment with kurarinone for 6 h suppressed the LPS-induced *Il-1β* and *iNos* mRNA expression in RAW264.7 cells. Furthermore, this suppressive effect was significantly rescued by the treatment with the HO-1 inhibitor TinPPIX. These results revealed that kurarinone suppresses the production of inflammatory mediators by activating the Nrf2/HO-1 pathway. The present study elucidated a new molecular mechanism by which kurarinone exerts its anti-inflammatory effects (Figure 5).

The majority of compounds that activate the KEAP1/Nrf2 pathway were electrophiles, which modify specific cysteine residues within KEAP1 [30]. Kurarinone induced KEAP1 reduction at both the mRNA and protein level. It is not clear how kurarinone reduces *KEAP1* mRNA levels. Interestingly, several microRNAs (miRNAs) (e.g., miR-141, miR-200a) were reported to target *KEAP1* mRNA by binding to its 3′-untranslated region sequence sites [31]. We speculated that the mechanism through which kurarinone reduces *KEAP1* mRNA might be that kurarinone represses *KEAP1* transcription, or that the induction of these miRNAs might reduce *KEAP1* mRNA. In addition, kurarinone reduced the KEAP1 protein levels at a later stage, but it is unclear whether kurarinone chemically modified the KEAP1 protein. Taguchi et al. showed that KEAP1 degradation was accelerated in electrophilic stress conditions [32]. Thus, the possibility exists that KEAP1 degradation might be promoted by chemically modifying the KEAP1 protein with kurarinone.

The in vivo antioxidant system centered on Nrf2 plays an important role in protecting cells from various stresses, including oxidative stress [33]. In response to stress, Nrf2 binds to the ARE present in gene promoters, with the small musculoaponeurotic fibrosarcoma (sMaf) as a partner, and induces antioxidant enzymes, such as HO-1, GPX, CAT, and NQO-1. In addition to HO-1, kurarinone induced the mRNA induction of the cystine transporter xCT, thioredoxin reductase 1 (TXNRD1), and SOD2 in PC3 cells. All of these genes are Nrf2 target genes [8], and kurarinone is considered to be a natural product-derived Nrf2 activator. We previously reported that kurarinone induced the activation of the activating transcriptional factor 4 (ATF4) by phosphorylating PKR-like ER kinase (PERK) [18]. ATF4 was also shown to induce antioxidant enzymes and was involved in the transcriptional regulation of the antioxidant enzymes described above [34]. Therefore, kurarinone might simultaneously activate intracellular Nrf2 and ATF4, inducing crosstalk in the antioxidant gene promoter [35]. However, the treatment with TG, which induces endoplasmic reticulum stress and activates ATF4, did not induce HO-1 (Figure 1G). Further studies are needed to clarify the induction of antioxidant enzymes through crosstalk between Nrf2 and ATF4.

Since oxidative stress is associated with many pathological conditions, such as cancer, neurodegenerative diseases, cardiovascular diseases, and metabolic diseases [36,37], clinical applications targeting the Nrf2 pathway are attracting increasing interest [38]. Sulforaphane contained in vegetables, such as broccoli, is a representative Nrf2 activator derived from natural products [39,40]. It was reported to suppress carcinogenesis caused by chemical substances, and the elimination of carcinogenic metabolic intermediates was shown to be promoted in vitro by the induction of drug-metabolizing enzymes, through the activation of Nrf2 [41]. Therefore, many clinical trials were conducted on the preventive effects of sulforaphane against cancer as well as cardiovascular diseases, neurodegenerative diseases, and diabetes [41].

Drug discovery research to activate the Nrf2 pathway is now actively conducted. Although bardoxolone methyl exhibits Nrf2-inducing, antioxidant, and anti-inflammatory activities and is expected to have clinical applications [42,43], clinical studies were discontinued due to toxicity [44]. Dimethyl fumarate is approved for the treatment of multiple sclerosis and is reported to activate Nrf2 [45,46]. In addition, tolvaptan, a vasopressin type 2 receptor antagonist, was shown to activate the Nrf2/HO-1 pathway via PERK [47]. Tolvaptan was proposed as a potential treatment for chronic kidney disease, and further research for clinical applications is ongoing. As described above, Nrf2 activation-based medicine is already at a practical level, and has the potential to offer new options for the treatment of many diseases.

In this study, we demonstrated that kurarinone represented antioxidant and anti-inflammatory properties via Nrf2 activation. Kurarinone-induced HO-1 expression was observed at a concentration of 20 μM. Although there are no reports in humans, there are few reports examining the pharmacokinetics of kurarinone in rats. Jiang et al. analyzed kurarinone levels in fluids and tissues of *S. flavescens* extract-treated rats (2.5 g/kg) [48]. After administration, kurarinone was more abundant in visceral organs and in the liver at a concentration close to 30 μM [48]. Therefore, it might be possible to actually achieve the antioxidant and anti-inflammatory effects of kurarinone in vivo. However, kurarinone was reported as a hepatotoxic constituent of *S. flavescens* in rats [48,49]. Although the detailed mechanism is not yet clarified, *S. flavescens* extract induced hepatic lipid accumulation and liver injury. In contrast, Yang et al. demonstrated that kurarinone showed no detectable toxicity in mice, since there were no significant effects on body weight, behavior, and appearance between the kurarinone-treated mice and the control [50]. Nrf2 is known to exert a protective effect in the pathogenesis of liver injury, both in vitro and in vivo [51,52]. Therefore, it is necessary to clarify the mechanism of hepatic injury caused by kurarinone. In addition, structure–activity relationship studies might reveal the structures required for Nrf2 activation and those that induce hepatotoxicity. It is important to create derivatives that exhibit antioxidant and anti-inflammatory activity, without hepatotoxicity.

## 5. Conclusions

In conclusion, kurarinone is expected to become a seed compound for new drugs that activate Nrf2. It might be used for clinical applications and has the potential to provide new therapeutic options for chronic inflammatory diseases. Further studies are needed to establish the precise mechanisms of action of kurarinone on the KEAP1/Nrf2 pathway.

## Figures and Tables

**Table 1:** Primer sets for RT-qPCR.

Gene	Primer Sequences	NCBI Accession Number
human *ACTB*	5′-TGGCACCCAGCACAATGAA-3′ 5′-CTAAGTCATAGTCCGCCTAGAAGCA-3′	NM_001101.4
human *CAT*	5′-CCATTATAAGACTGACCAGGGC-3′ 5′-AGTCCAGGAGGGGTACTTTCC-3′	NM_001752.3
human *GPX1*	5′-CAGTCGGTGTATGCCTTCTCG-3′ 5′-GAGGGACGCCACATTCTCG-3′	NM_000581.3
human *GPX2*	5′-CCCCTACCCTTATGATGACC-3′ 5′-GTTGATGGTTGGGAAGGTG-3′	NM_002083.3
human *GPX3*	5′-CGGGGACAAGAGAAGTCG-3′ 5′-CCCAGAATGACCAGACCG-3′	NM_002084.4
human *GPX4*	5′-GAGTTTTCCGCCAAGGACATCGA-3′ 5′-GGTCGACGAGCTGAGTGTAGTTT-3′	NM_002085.4
human *GSR*	5′-ATGATCAGCACCAACTGCAC-3′ 5′-CGACAAAGTCTTTTTAACCTCCTT-3′	NM_000637.4
human *GSS*	5′-AAGACACTCGTGATGAACAAG-3′ 5′-AGAGGAATGACAAATACAGAGGAT-3′	NM_000178.3
human *HO-1*	5′-ATGGCCTCCCTGTACCACATC-3′ 5′-TGTTGCGCTCAATCTCCTCCT-3′	NM_002133.2
human *KEAP1*	5′-ATTGGCTGTGTGGAGTTGC-3′ 5′-CAGGTTGAAGAACTCCTCTTGC-3′	NM_012289.3
human *NQO-1*	5′-ATCCTGCCGAGTCTGTTCTG-3′ 5′-AGGGACTCCAAACCACTGC-3′	NM_000903.2
human *Nrf2*	5′-CTTTTGGCGCAGACATTCC-3′ 5′-AAGACTGGGCTCTCGATGTG-3′	NM_006164.4
human *PRDX1*	5′-AGGCCTTCCAGTTCACTGAC-3′ 5′-CAGGCTTGATGGTATCACTGC-3′	NM_002574.3
human *SOD1*	5′-TGGTTTGCGTCGTAGTCTCC-3′ 5′-CTTCGTCGCCATAACTCGCT-3′	NM_000454.4
human *SOD2*	5′-GGAAGCCATCAAACGTGACTT-3′ 5′-CCCGTTCCTTATTGAAACCAAGC-3′	NM_000636.3
human *SOD3*	5′-GGTGCAGCTCTCTTTTCAGG-3′ 5′-AACACAGTAGCGCCAGCAT-3′	NM_003102.2
human *TXN*	5′-GCCTTTCTTTCATTCCCTCTC-3′ 5′-GCTTTTCCTTATTGGCTCCAG-3′	NM_003329.3
human *TXNRD1*	5′-TGTTGGAGCATCCTATGTCG-3′ 5′-TCAAATCCTCTAAGAAGAATGGACC-3′	NM_182729.2
human *xCT*	5′-TCCTGCTTTGGCTCCATGAACG-3′ 5′-AGAGGAGTGTGCTTGCGGACAT-3′	NM_014331.3
mouse *Actb*	5′-GGCTGTATTCCCCTCCATCG-3′ 5′-CCAGTTGGTAACAATGCCATGT-3′	NM_007393.5
mouse *Ho-1*	5′-ACAGAGGAACACAAAGACCAG-3′ 5′-GTGTCTGGGATGAGCTAGTG-3′	NM_010442.2
mouse *Il-1β*	5′-GCAACTGTTCCTGAACTCAACT-3′ 5′-ATCTTTTGGGGTCCGTCAACT-3′	NM_008361.4
mouse *iNos*	5′-GGCAGCCTGTGAGACCTTTG-3′ 5′-TGCATTGGAAGTGAAGCGTTT-3′	NM_010927.4
mouse *Nrf2*	5′-CCCAGCAGGACATGGATTTGA-3′ 5′-AGCTCATAGTCCTTCTGTCGC-3′	NM_010902.4

**Table 2:** Primer sets for ChIP-qPCR.

Gene	Primer Sequences	NCBI Accession Number
human *HO-1* p1	5′-GCTGAGTCGCGATTTCCTCAT-3′ 5′-GAGGCTTCTGCCGTTTTCTA-3′	NC_000022.11
human *HO-1* p2	5′-CCCTGCTGAGTAATCCTTTCC-3′ 5′-TTAAACCTGGAGCAGCTGGA-3′	NC_000022.11
human *HPRT1-*int1	5′-TGTTTGGGCTATTTACTAGTTG-3′ 5′-ATAAAATGACTTAAGCCCAGAG-3′	NC_000023.11
